# Intensive blood pressure control is associated with improved patient and graft survival after renal transplantation

**DOI:** 10.1038/s41598-019-46991-2

**Published:** 2019-07-19

**Authors:** Nikolaos Pagonas, Frederic Bauer, Felix S. Seibert, Maximilian Seidel, Peter Schenker, Stylianos Kykalos, Michael Dürr, Petra Reinke, Nina Babel, Richard Viebahn, Timm H. Westhoff

**Affiliations:** 1 Medical Department I, University Hospital Marien Hospital Herne, Ruhr-University of Bochum, Bochum, Germany; 2 Department of Cardiology, Brandenburg Medical School, University Hospital Brandenburg, Brandenburg, Germany; 3 Department of General, Visceral and Transplant Surgery, University Hospital Knappschaftskrankenhaus Bochum, Ruhr-University of Bochum, Bochum, Germany; 4 Department of Nephrology, Charité – Universitätsmedizin Berlin, Berlin, Germany

**Keywords:** Nephrology, Chronic kidney disease

## Abstract

Based on data of the SPRINT trial, American hypertension guidelines recently reduced the blood pressure goal from 140/90 mmHg to 130/80 mmHg for subjects with chronic kidney disease (CKD), whereas European guidelines recommend a systolic blood pressure (SBP) of 130–139 mmHg. The present analysis investigates whether a SBP < 130 mmHg is associated with an additional benefit in renal transplant recipients. We performed a retrospective analysis of 815 renal transplant recipients who were stratified according to mean office SBP values < 130 mmHg, 130–139 mmHg or ≥140 mmHg. Patient and graft survival was defined as composite endpoint, follow-up was limited to 120 months. Mean SBP of the follow-up was significantly associated with the composite endpoint (n = 218) with better survival for a SBP < 130 mmHg and 130–139 mmHg compared to ≥140 mmHg (p < 0.001). The differences in the combined endpoint remained significant in Cox regression analysis adjusted for age, gender and eGFR (p = 0.007, HR = 0.58, 95%CI = 0.41–0.53), but not for graft survival alone. Renal transplant recipients with SBP < 130 mmHg had a lower mortality than those with the conservative blood pressure goal <140 mmHg. These data suggest that the new AHA BP targets are safe for renal transplant recipients and – with all limitations of a retrospective analysis - might even be associated with improved outcome.

## Introduction

Cardiovascular events are the leading cause of death and a major reason for graft loss after renal transplantation^1,2^. The cardiovascular risk of the transplant population exceeds the risk of the general population by far^3^. Hypertension has a prevalence of 80% in renal transplant recipient and represents one of the crucial determinants for cardiovascular events^4^. The optimal blood pressure (BP) target for this population remains elusive. To date, there are no randomized trials on the management of hypertension after kidney transplantation^5^. Current guideline recommendations are conflicting. The 2009 KDIGO guidelines on renal transplantation recommend a systolic blood pressure (SBP) goal of <130 mmHg. This recommendation was based on former hypertension guidelines of the general population suggesting this goal for chronic kidney disease (CKD)^6^. Since that time several international hypertension guidelines - including the European and the American ones – liberalized BP targets for non-proteinuric CKD to <140 mmHg^7,8^.

The SPRINT trial led to a reversal of the liberalization of BP targets in CKD. A mean SBP < 130 mmHg was associated with a substantial reduction of cardiovascular and overall mortality compared to a conventional BP management in a cardiovascular high risk population^9^. Interestingly, 28% of the SPRINT population suffered from CKD. In this subgroup intensive blood pressure control led to a 27% reduction of mortality. In November 2017, the American Heart Association (AHA) therefore adapted their blood pressure targets. For subjects at high cardiovascular risk – including CKD – the new SBP target is <130 mmHg^10^. The new guidelines from the European Society of Hypertension were published after the AHA guidelines in 2018 and recommend a systolic target blood pressure of 130–139 mmHg and a diastolic blood pressure <80 mmHg for CKD^11^. Thus, there is currently no consensus on the ideal blood pressure in subjects with impaired kidney function and thereby renal transplant recipients (3).

It is not clear, which guidelines should be regarded suitable for renal transplant recipients and whether these subjects benefit from an intensive BP control. The aim of the present retrospective analysis of three transplant centers in Germany is to investigate whether an intensive BP control is associated with a better graft and patient survival than conservative BP control.

## Results

### Transplant characteristics

858 subjects were screened for inclusion. 43 subjects were excluded for reasons of incomplete BP documentation. 815 patients were enrolled and stratified according to mean SBP over the complete follow-up (Supplementary Fig. S1). Epidemiological and transplant related data is summarized in Tables 1 and 2. Mean age of the study population at the time of transplantation was 50 (17–89) years. Patients with a mean SBP > 140 mmHg were significantly older than the other groups (54.8 vs. 46.4 and 49.4 years, p < 0.001). Data on proteinuria at 12 months after transplantation were available in 258 patients. Mean proteinuria at 12 months was 192.2 ± 556.6 mg/l (data available in 258 patients). The most frequent cause of end stage renal disease was glomerulonephritis (37.7%), followed by cystic kidney disease (15.6%), interstitial nephritis (9.7%) and diabetes mellitus (6.0%). In 12.5% of the patients the cause of the ESRD was unknown. Patients in the lowest BP group (<130 mmHg) had more often a live donor transplantation (19.2%, p = 0.006) from a younger donor (mean donor age = 45.3 years, p < 0.001). Groups did not significantly differ in terms of time on dialysis, cause of kidney disease, donor age and gender, comorbidities, and body mass index. A triple therapy of calcineurininhibitor (48.8% tacrolimus, 51.2% cyclosporine), mycophenolic acid, and a steroid constituted the most frequent immunosuppressive regimen in all the three groups reaching the highest prevalence in the group with highest SBP levels (p < 0.001, Table 2). The number of acute rejection episodes did not differ between the groups during follow-up. 570 of 815 patients had no episodes, 189 patients had one rejection episode and 56 patients had two or more episodes of acute rejections during the period of 120 months. Mean BP at 12 months was 134.3 ± 13.2/ 79.2 ± 7.7 mmHg. There were no significant differences in the use of any individual class of immunosuppressive drugs within the three groups (p > 0.05 each).

**Table 1:** Characteristics of study population.

	Total (n = 858)	Mean systolic blood pressure during 120 months (n = 815)			p
		<130 mmHg (n = 286)	130–139 mmHg (n = 262)	≥140 mmHg (n = 267)	
Male	516 (60.1%)	150 (52.4%)	166 (63.4%)	169 (63.3%)	0.001
Age (years, low-high)	50.0 (17–89)	46.4 (17–89)	49.4 (22–78)	54.8 (26–77)	<0.001
Body mass index (kg/m^2^)	25.2 ± 4.2	24.2 ± 4.2	26.2 ± 4.4	25.5 ± 3.7	0.06
Time on dialysis (months, min-max)	65.9 (0–382)	66.7 ± 47.5	67.1 ± 41.9	66.1 ± 44.9	0.96
***Concomitant diseases***					
Hypertension	776 (90.5%)	261 (91.3%)	231 (88.5%)	242 (90.6%)	0.53
Diabetes mellitus	177 (20.7%)	53 (18.5%)	47 (18.0%)	63 (23.6%)	0.20
Coronary heart disease	151 (17.7%)	48 (16.8%)	44 (17.0%)	53 (19,9%)	0.59
Hyperlipidaemia	271 (31.7%)	88 (30.9%)	77 (29.6%)	85 (31.8%)	0.86

Continuous data are presented as mean and standard deviation and were tested for statistically significant differences by unpaired t-tests. Categorical data (gender, live donation) were compared by Fisher’s exact test. P < 0.05 was regarded statistically significant.

**Table 2:** Renal and transplant characteristics.

	Total (n = 858)	Mean systolic blood pressure during 120 months (n=815)			p
		<130 mmHg (n = 286)	130–139 mmHg (n = 262)	≥140 mmHg (n = 267)	
*General characteristics*					
Live donor transplantation	117 (13.9%)	54 (19.2%)	33 (12.7%)	26 (9.9%)	0.006
Donor sex male	424 (54.2%)	142 (54.8%)	132 (55.2%)	134 (55.1%)	0.99
Donor age (years)	49.5 (4–88)	45.3 (4–76)	51.0 (10–88)	53.5 (9–84)	<0.001
Donor eGFR (ml/min*1.73 m^2^)	70.0 ± 44.8	73.8 ± 38.6	66.9 ± 42.7	69.5 ± 43.1	0.71
eGFR (ml/min*1.73 m^2^) after 12 months	41.3 ± 19.5	48.2 ± 19.8	43.4 ± 16.8	37.9 ± 17.5	<0.001
Proteinuria after 12 months (mg/l)	192 ± 555	184 ± 777	176 ± 358	230 ± 454	0.82
Rejection during follow-up	245	80	88	77	0.62
***Immunsuppression (month 12)***					
Triple immunsuppression	704 (83.6%)	216 (76.3%)	227 (88.7%)	248 (94.3%)	<0.001
Mono/dual immunsuppression	138 (16.4%)	67 (23.7%)	29 (11.3%)	15 (5.75)	
Steroids	830 (98.2%)	281 (98.6%)	251 (97.7%)	259 (98.9%)	0.53
Mycophenolic acid	692 (81.9%)	230 (80.7%)	217 (84.4%)	230 (87.8%)	0.08
Cyclosporine	343 (40.6%)	114 (40%)	100 (38.9%)	121 (46.2%)	0.19
Tacrolimus	412 (48.8%)	131 (46.0%)	131 (51%)	132 (50.45)	0.44
Other	73 (8.7%)	17 (6.1%)	31 (12.1%)	21 (7.9%)	0.31

Data on proteinuria available only in 258 patients. Data on other parameters always available in >800 of the patients.

### Blood pressure during follow-up

**Figure 1:**
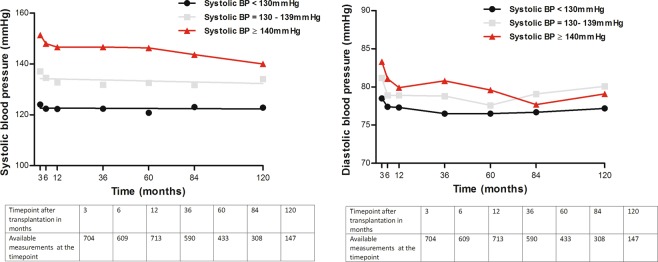
Course of systolic and diastolic blood pressure over the follow-up period in dependence of mean systolic blood pressure (SBP) < 130 mmHg, 130–139 mmHg, or ≥140 mmHg over a period of 120 months.

Patients were followed-up for a median of 83.5 months (13–120 months). Mean BP was 133.2 ± 11.8 mmHg in the overall follow-up period. As presented by Table 3 both stratification approaches led to significantly different BP values in the three groups. Pulse pressure differed significantly among the groups as well (p < 0.001). Figure 1 and Table 3 present the course of the systolic and diastolic BP during the follow-up. BP differed highly significantly at the beginning of the analysis (p < 0.001). This difference did not reach statistical significance anymore at the end of the observation (e.g. 84 and 120 months after transplantation, p > 0.05).

**Table 3:** Hemodynamic data of the study population after stratification for systolic blood pressure (BP).

	Overall population (n = 815)	Systolic BP < 130 mmHg (n = 286)	Systolic BP 130–139 mmHg (n = 262)	Systolic BP ≥ 140 mmHg (n = 267)	p
Number of measurements	3454	1322	1277	855	
Median number of antihypertensive drugs (month 12)	3 (0–6)	2 (0–6)	3 (0–6)	3 (0–6)	<0.001
**Complete follow-up**					
Systolic BP (mmHg)	133.2 ± 11.8	122.3 ± 5.8	133.7 ± 2.8	148.2 ± 8.7	<0.001
Diastolic BP (mmHg)	78.7 ± 6.6	76.8 ± 5.8	79.2 ± 6.0	80.6 ± 7.5	<0.001
Pulse pressure (mmHg)	54.6 ± 11.7	45.5 ± 6.2	54.5 ± 6.6	67.6 ± 10.6	<0.001
**First 12 months post transplant**					
Systolic BP (mmHg)	134.2 ± 13.2	121.1 ± 6.9	133.7 ± 2.9	148.6 ± 8.8	<0.001
Diastolic BP (mmHg)	79.2 ± 7.7	76.5 ± 7.2	79.9 ± 7.1	81.5 ± 7.9	<0.001
Pulse pressure (mmHg)	54.9 ± 12.7	44.6 ± 7.3	53.8 ± 7.4	67.1 ± 10.9	<0.001

Intergroup differences were examined by analysis of variance (ANOVA); data presented as mean ± standard deviation of BP values during follow-up.

### Survival analysis

**Figure 2:**
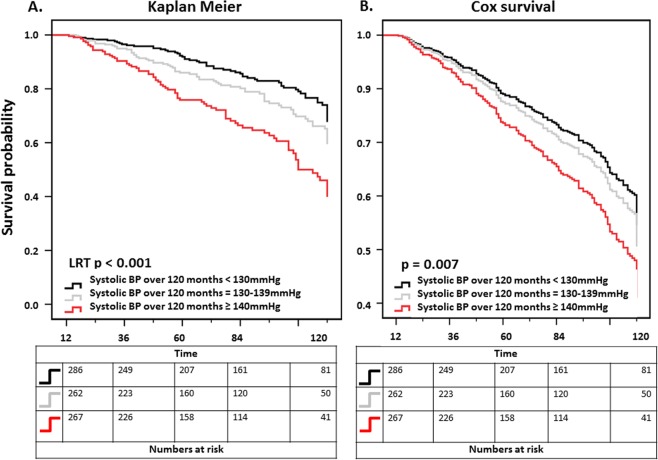
Kaplan Meier curve (**A**) and Cox survival curve (**B**, adjusted for age, eGFR at 12 months, gender, donor age and number of immunosuppressive drugs) for the composite endpoint of patient and graft survival in dependence of mean systolic blood pressure (SBP) < 130 mmHg, 130–139 mmHg, or ≥140 mmHg over a period of 120 months; LRT: log rank test. In Cox analysis p refers to the statistical analysis of the lowest group (SBP < 130 mmHg) compared to the reference group (SBP ≥ 140 mmHg).

During the follow-up a total of 118 patients returned to dialysis and 110 patients died. Kaplan Meier analysis revealed significantly different cumulative survival curves in dependence of BP. The lowest SBP was associated with best survival rates, the highest BP with lowest survival rates (p < 0.001). The composite endpoint of patient and graft survival differed significantly among the groups in an analogous manner (p < 0.001, Fig. 2A). Cumulative graft survival was inversely associated with SBP as well. In analogy to the patient survival analysis, lowest BP was associated with best cumulative survival (p < 0.001). Posthoc log-rank testing for those subjects with 130–139 mmHg vs. < 130 mmHg still revealed significant differences in the composite endpoint (p = 0.03) and death (p = 0.04) but not in graft survival (p = 0.39). Figure 2B shows the cox survival curves adjusted for age, gender, eGFR 12 months after transplantation, donor age and immunosuppressive drugs demonstrating a persistently better cumulative survival for the lower blood pressure group against patients with SBP > 140 mmHg (reference group) regarding the primary composite endpoint (p = 0.007, HR = 0.58, 95%CI = 0.41–0.53). Mortality alone was also lower in patients with lower SBP (p = 0.03, HR = 0.53, 95%CI = 0.32–0.88). By comparing only the group of patients with SBP = 130–139 mmHg to the reference group (SBP ≥ 140 mmHg) a trend for a better survival for the composite end point was seen (p = 0.08, HR = 0.66, 95%CI = 0.41–1.0). The benefit in graft survival, seen in the Kaplan Meier analysis, was not statistically significant in the cox analysis (p = 0.09).

In a second analysis, we assessed whether the mean SBP of the first 12 months predicted future patient and graft survival. Stratification was performed using the same BP intervals. In accordance with the stratification by 120 months BP values, subjects with a mean SBP < 130 mmHg over the first 12 months presented the highest survival with regard to the primary composite endpoint of patient and graft survival followed by the group with a SBP 130–139 mmHg (p < 0.001). Outcome was worst, if mean SBP was ≥140 mmHg in the first 12 months after transplantation. Supplementary Fig. S2 shows the Kaplan Meier analyses with a significant difference in cumulative patient survival (p < 0.001). Subjects with a mean SBP < 130 mmHg presented the highest survival for all-cause mortality (p < 0.001) and graft loss (p = 0.03). In the Cox analysis the survival benefit was not statistically significant (p = 0.49, Supplementary Fig. S2).

## Discussion

“It is unlikely that there will be randomized controlled trials in kidney transplant recipients to determine whether blood pressure lowering reduces cardiovascular disease events, or prolongs patient or graft survival.” Almost 10 years after the publication of this statement in the KDIGO guidelines on renal transplantation, this prediction is still true^12^. A well performed retrospective analysis – with all its inherent limitations - in a large cohort of subjects is therefore currently the best way to address this question of daily clinical interest.

Former retrospective analyses have convincingly shown that a SBP < 140 mmHg is associated with improved patient and graft survival^13^. The present study shows for the first time that a SBP target <130 mmHg is associated with an even better outcome. In all Kaplan Meier and Cox regression analyses cumulative survival regarding both the primary composite and the individual secondary endpoints was best in the group with strict BP control (<130 mmHg), followed by conservative BP control (130–139 mmHg) and worst in those subjects with uncontrolled hypertension. The post-hoc log-rank testing of the two lower BP groups proved that the additional benefit on the composite endpoint was statistically significant suggesting that the new blood pressure target of the AHA guidelines might be suitable for renal transplant recipients as well. Moreover, this analysis uncovered that this additional benefit is primarily driven by the reduction of mortality and not the reduction of graft loss. This finding resembles the outcome of the SPRINT CKD population. Intensive BP control resulted in a significant reduction of mortality but only to a trend in renal endpoints like doubling of creatinine or initiation of dialysis^9,14^. The SPRINT CKD population had a very low grade of proteinuria with a mean urinary albumin-creatinine ratio of only 13 mg/g^14^. Several trials in non-transplant CKD populations have demonstrated that intensive compared to conservative BP lowering does only provide additional GFR-sparing benefits in proteinuric CKD.

The African American Study of Kidney Disease and Hypertension (AASK) trial had a large impact on the liberalization in the CKD population. A blood pressure target <130 mmHg did not result in a better conservation of renal function than a goal <140 mmHg over a period of 3–6 years in a population of almost 1100 subjects with CKD. At closer view, however, proteinuric patients actually did benefit from the intensive blood pressure control, whereas only non-proteinuric subjects did not. The AASK trial thereby confirmed the historical findings of the Modification of Diet in Renal Disease (MDRD) study^15^. In summary, data of the non-transplant CKD population suggest a convincing cardiovascular benefit by intensive BP control, whereas the additional effects on slowing the progression of renal disease are restricted to proteinuric subjects. The present population had a low level of proteinuria (>200 mg at month 12), which may explain why the trend to improve graft survival did not reach statistical significance.

As illustrated by Fig. 2 BP decreases within the first 12 months post transplant, which can be attributed to several aspects: Glomerular filtration usually increases in the first weeks after transplantation, whilst vasoactive immunosuppression like calcineurin inhibitors and steroids are continuously reduced. Moreover, antihypertensive medication is frequently paused at the timepoint of transplant and consecutively reimplemented in the period thereafter.

But is BP indeed the chicken or could it be the egg? As demonstrated in SPRINT, a lower BP can result in a reduction of mortality. A “better” allograft function, however, could result in lower BP as well. A retrospective analysis is never able to prove causality. In order to minimize this possibility, adjustment by baseline eGFR was performed in the Cox regression. Noteworthy, the benefit in both the composite endpoint and mortality remained, whereas only the benefit in graft survival lost statistical significance. These data indicate a major “chicken” role of BP control on patient survival and a minor causal role on graft survival. The second cox regression model additionally including live donor transplantation and donor age as covariates still showed significant differences among the three groups. Thus, these differences are unlikely to be explained by the heterogeneity of groups.

Our data are in line with previous observations in the literature: Thus, a 10 mmHg increase of SBP was associated with an increase in the risk of graft loss by 12%^16^. The Collaborative Transplant Study revealed an association between BP and outcome as well. The present study, however, differs from these historical ones in two crucial aspects. First, it compares a SBP < 130 with <140 mmHg for the first time. Second, it contains an adjustment for several parameters including baseline eGFR, thus making a causal relationship between BP control and improved outcome very probable.

The second mode of stratification showed that the mean SBP of the first 12 months predicts outcome. In analogy with the first analysis, there were significant differences in both the primary and the two individual secondary endpoints death and graft survival. Still, a SBP < 130 mmHg performed best. This finding underlines the importance of BP control early after transplantation. Interestingly, one-year BP was recently identified as a major risk factor for deteriorating kidney function after heart or liver transplantation as well^17^. In analogy to the SPRINT trial, we did primarily focus on systolic and not on diastolic BP in the present analysis, since diastolic BP decreases with increasing arterial stiffness.

What are the consequences of these data for clinical practice? In 2004 50% of the American transplant recipients did not reach the BP goal <140 mmHg^16^. The present study population performed somewhat better with two thirds reaching this goal. Nevertheless, is it reasonable to call for a more challenging BP goal, when one third of our patients still do not reach the conservative one? Yes indeed, since “what is better is the enemy of what is good.” Moreover, a more ambitious BP goal is likely to increase the rate of subjects reaching at least <140 mmHg.

The study is primarily limited by its retrospective character. The adjustments for eGFR in cox regression and the confirmatory Kaplan Meier analysis using a second stratification approach attenuate this limitation as far as possible. Nevertheless, a retrospective analysis can never prove causality. Moreover, data on proteinuria were not available for the complete study population.

The present retrospective study analyzed the application of the new hypertension guidelines to renal transplant recipients for the first time. Renal transplant recipients who achieve a mean SBP < 130 mmHg had a significantly lower mortality than with the conservative goal <140 mmHg. This finding remained unchanged after adjustment for baseline allograft function. These data suggest that the new BP targets are safe for renal transplant recipients and – with all limitations of a retrospective analysis - might even be associated with improved outcome.

## Methods

### Study design and protocol

Patients who underwent kidney transplantation at two transplant centres of the Charité – Universitätsmedizin Berlin, Germany (Campus Benjamin Franklin and Campus Virchow Klinikum) and at the University Hospital Knappschaftskrankenhaus Bochum between 1997 and 2011 were screened for inclusion in this retrospective study. Patients were selected from the outpatient clinics of these transplantation centres. Data for analysis were collected from patients undergoing routine examinations based on clinical indications in a retrospective manner, thus making an approval from the local ethical board unnecessary.

Inclusion criteria were renal transplantation between 1997 and 2011 and documented follow-up examinations including laboratory and BP values in the outpatient clinic for at least 12 months after transplantation with at least 4 documented visits in the outpatient clinic. Data were documented from single measurements of 7 visits during follow-up: 3, 6, 12, 36, 60, 84 and 120 months after transplantation.

### Measures, variables and definitions

BP measurements took place in the outpatient clinics of the three transplant centers according to each center’s standard. Both manual auscultatory BP measurement and oscillometric blood pressure measurement by automated devices were applied. Measurements took place in separate rooms of the outpatient clinic by a nurse, who stayed in the room. The nurse usually refrained from repeated measurements. Thus, the analysis was based on single office BP values. The population was characterized for epidemiological parameters, cardiovascular comorbidities, renal disease, and transplant related parameters. The presence of a concomitant disease was presumed if such a diagnosis (e.g. hypertension or coronary artery disease) was found in the medical history of the patient. Antihypertensive or antidiabetic medication allowed to categorize a patient as having hypertension or diabetes mellitus. Graft loss was defined as reinitiating any type of renal replacement therapy.

Death or graft loss was defined as primary composite endpoint. Subjects were stratified according to their SBP in two statistical approaches. First, the stratification was based on the mean SBP of the above mentioned visits during the complete follow-up (3–120 months). The second analysis investigated the impact of the first year’s BP on outcome parameters. Hence, mean SBP of the first 12 months posttransplant was used for stratification. The timepoint of 12 months was selected since graft function, antihypertensive and immunosuppressive medication have usually reached a “steady state” at this time. Subjects were divided into three groups: The first one achieving the new AHA goal SBP < 130 mmHg, the second one fulfilling the European guidelines (SBP 130–139 mmHg), and the third one failing to reach the criteria of any of these guidelines (SBP ≥ 140 mmHg). Data about the diastolic blood pressure were documented and analyzed but patients were divided in the groups based only on SBP.

### Statistical analysis

Continuous data are presented as mean ± standard deviation, discrete data as median and range. Data was tested for normal distribution by the Kolmogorov-Smirnov test. Comparison of categorical parameters was performed by Fisher’s exact test and Pearson-Χ^2^-test. Intergroup differences of BP and pulse pressure were examined by analysis of variance (ANOVA). Cumulative patient and graft survival was calculated by Kaplan Maier analyses after stratification of patients by their mean SBP over the follow-up period. Cox regression analyses were performed for the primary composite endpoint and the individual secondary endpoints. They were adjusted for all the parameters that showed a significant intergroup difference at baseline (age, eGFR at 12 months, gender, donor age and number of immunosuppressive drugs). Cox’s proportional hazards model was used as multivariate analysis to estimate hazard ratios (HR) with 95% confidence intervals (CI) for the study outcomes. Kaplan Maier analyses were repeated using a stratification by mean SBP of the first 12 months posttransplant. Post-hoc log- rank test was used to determine significant differences. P < 0.05 was regarded statistically significant. All statistical analyses were done using SPSS Statistics 21 (SPSS Inc, Chicago, Illinois, USA) and Prism 6 (GraphPad Software, La Jolla, California, USA).

## Supplementary information

Supplement

## References

[1] Ojo AO (2000). Long-term survival in renal transplant recipients with graft function. Kidney Int.

[2] Kasiske BL (2001). Epidemiology of cardiovascular disease after renal transplantation. Transplantation.

[3] Aziz F, Clark D, Garg N, Mandelbrot D, Djamali A (2018). Hypertension guidelines: How do they apply to kidney transplant recipients. Transplantation reviews.

[4] Ojo AO (2006). Cardiovascular complications after renal transplantation and their prevention. Transplantation.

[5] Halimi JM (2017). Optimizing hypertension management in renal transplantation: a call to action. Nephrology, dialysis, transplantation: official publication of the European Dialysis and Transplant Association - European Renal Association.

[6] Chobanian AV (2003). The Seventh Report of the Joint National Committee on Prevention, Detection, Evaluation, and Treatment of High Blood Pressure: the JNC 7 report. JAMA.

[7] James PA (2014). 2014 evidence-based guideline for the management of high blood pressure in adults: report from the panel members appointed to the Eighth Joint National Committee (JNC 8). JAMA.

[8] Mancia G (2013). 2013 ESH/ESC Guidelines for the management of arterial hypertension: the Task Force for the management of arterial hypertension of the European Society of Hypertension (ESH) and of the European Society of Cardiology (ESC). J Hypertens.

[9] Wright JT, Whelton PK, Reboussin DM (2016). A Randomized Trial of Intensive versus Standard Blood-Pressure Control. N Engl J Med.

[10] Whelton PK, Williams B (2018). The 2018 European Society of Cardiology/European Society of Hypertension and 2017 American College of Cardiology/American Heart Association Blood Pressure Guidelines: More Similar Than Different. JAMA.

[11] Williams B (2018). 2018 ESC/ESH Guidelines for the management of arterial hypertension: The Task Force for the management of arterial hypertension of the European Society of Cardiology and the European Society of Hypertension: The Task Force for the management of arterial hypertension of the European Society of Cardiology and the European Society of Hypertension. Journal of hypertension.

[12] McLain TA (2015). Development of an Anaerobic Sprint Running Test Using a Nonmotorized Treadmill. Journal of strength and conditioning research.

[13] Opelz G, Dohler B (2005). & Collaborative Transplant, S. Improved long-term outcomes after renal transplantation associated with blood pressure control. Am J Transplant.

[14] Cheung AK (2017). Effects of Intensive BP Control in CKD. J Am Soc Nephrol.

[15] Peterson JC (1995). Blood pressure control, proteinuria, and the progression of renal disease. The Modification of Diet in Renal Disease Study. Ann Intern Med.

[16] Kasiske BL (2004). Hypertension after kidney transplantation. Am J Kidney Dis.

[17] Morath C, Opelz G, Dohler B, Zeier M, Susal C (2018). Influence of Blood Pressure and Calcineurin Inhibitors on Kidney Function After Heart or Liver Transplantation. Transplantation.

