# Supplementary material for: Intensive blood pressure control is associated with improved patient and graft survival after renal transplantation
Source: Sci Rep. 2019 Jul 19;9:10507. doi: 10.1038/s41598-019-46991-2 (PMC6642181; doi:10.1038/s41598-019-46991-2)

**Intensive blood pressure control is associated with improved patient and graft survival after renal transplantation**

Nikolaos Pagonas, Frederic Bauer, Felix S. Seibert, Maximilian Seidel, Peter Schenker, Stylianos Kykalos, Michael Dürr, Petra Reinke Nina Babel, Richard Viebahn, Timm H. Westhoff

**Supplementary Figure S1:** Flowchart of the study

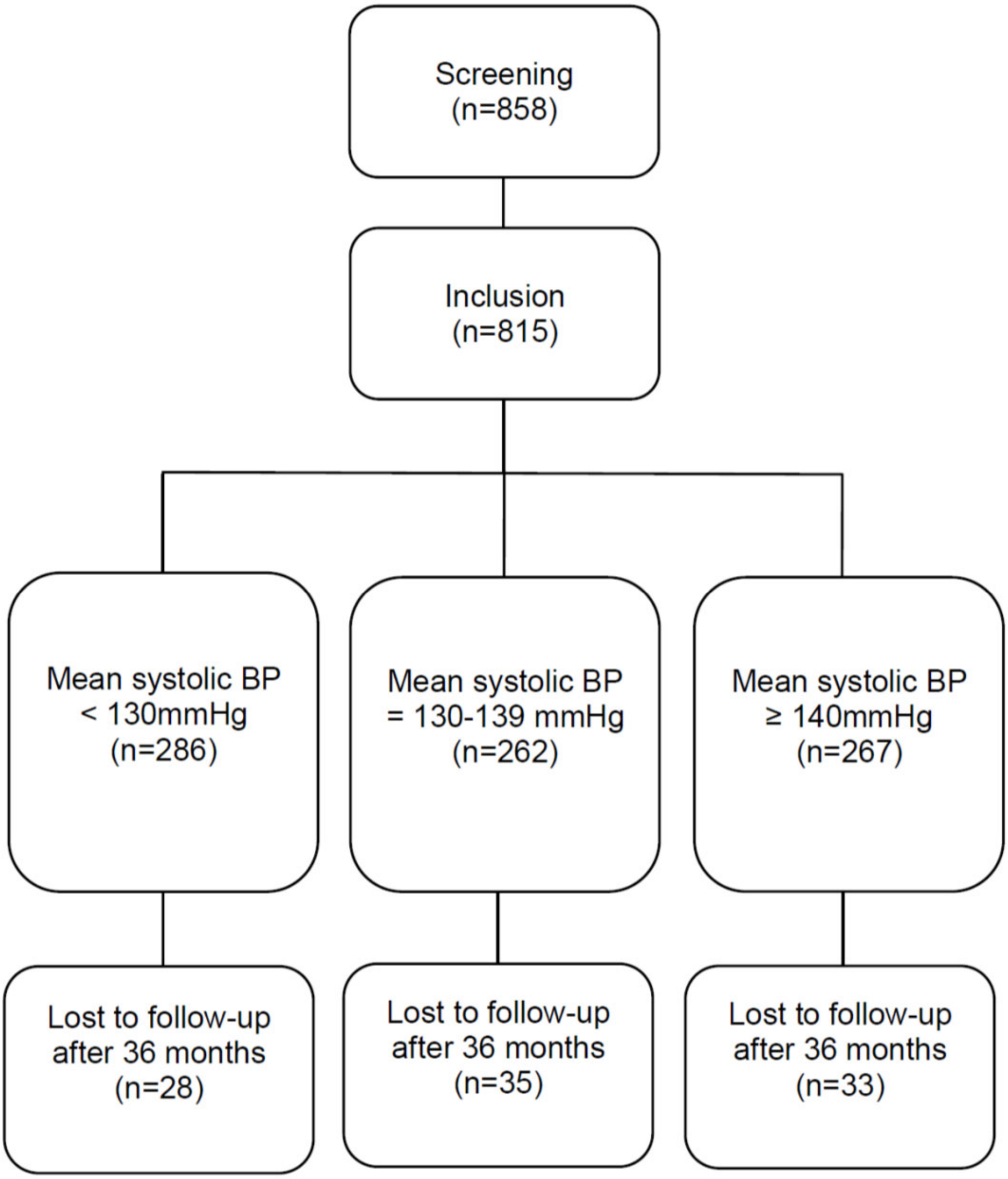

**Supplementary Figure S2:** Kaplan Meier curve (A) and Cox survival curve (B, adjusted for age, eGFR at 12 months, gender, donor age and number of immunosuppressive drugs) for the composite endpoint of patient and graft survival in dependence of mean systolic blood pressure (SBP) <130 mmHg, 130-139 mmHg, or ≥140 mmHg over a period of 12 months; LRT: log rank test. In cox analysis p refers to the statistical analysis of the lowest group (SBP < 130mmHg) compared to the reference group (SBP ≥140 mmHg).

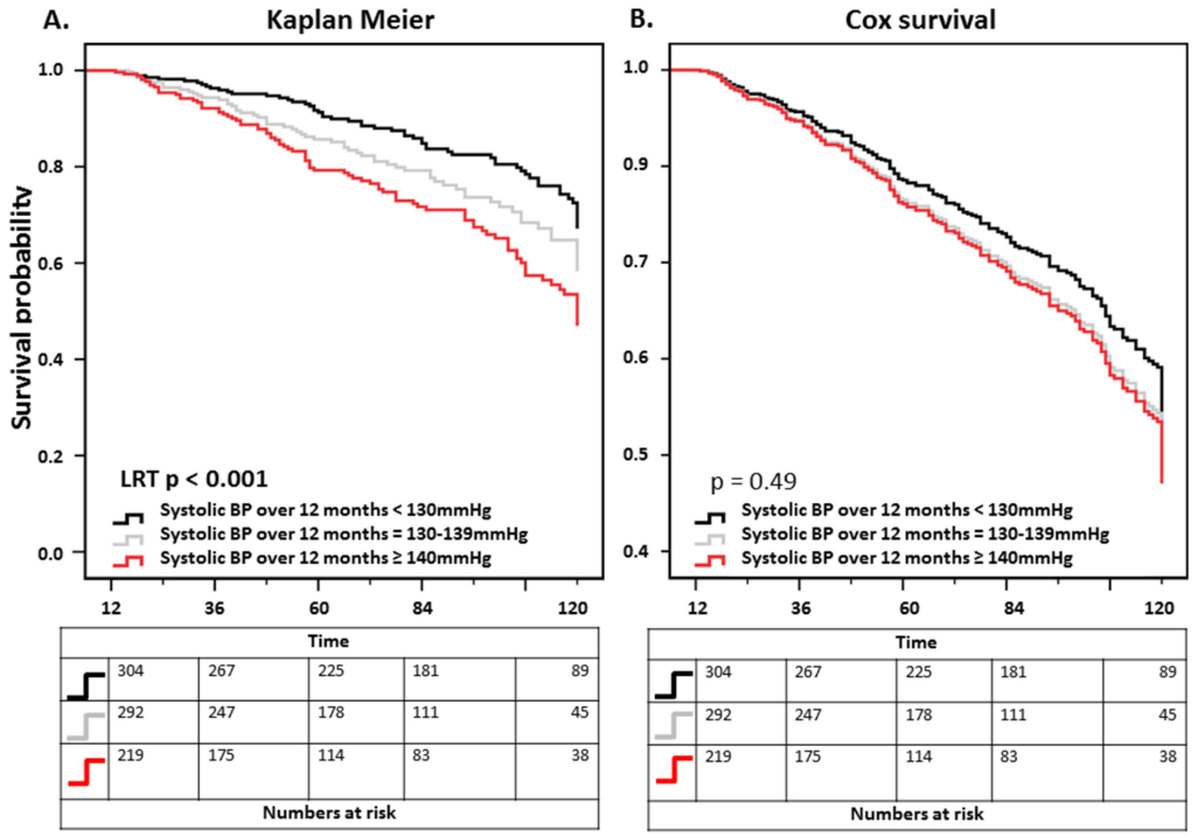

Supplement: Supplementary file 1 — Supplement [file 41598_2019_46991_MOESM1_ESM.pdf]
